# HIV-1 diversity among young women in rural South Africa: HPTN 068

**DOI:** 10.1371/journal.pone.0198999

**Published:** 2018-07-05

**Authors:** Mariya V. Sivay, Sarah E. Hudelson, Jing Wang, Yaw Agyei, Erica L. Hamilton, Amanda Selin, Ann Dennis, Kathleen Kahn, F. Xavier Gomez-Olive, Catherine MacPhail, James P. Hughes, Audrey Pettifor, Susan H. Eshleman, Mary Kathryn Grabowski

**Affiliations:** 1 Department of Pathology, Johns Hopkins University School of Medicine, Baltimore, MD, United States of America; 2 Fred Hutchinson Cancer Research Center, Seattle, WA, United States of America; 3 FHI360, Durham, NC, United States of America; 4 Carolina Population Center, University of North Carolina at Chapel Hill, Chapel Hill, NC, United States of America; 5 Division of Infectious Diseases, University of North Carolina at Chapel Hill, Chapel Hill, NC, United States of America; 6 MRC/Wits Rural Public Health and Health Transitions Research Unit (Agincourt), School of Public Health, Faculty of Health Sciences, University of the Witwatersrand, Johannesburg, South Africa; 7 School of Health and Society, University of Wollongong, New South Wales, Australia; 8 University of Washington, Seattle, WA, United States of America; 9 Department of Epidemiology, University of North Carolina at Chapel Hill, Chapel Hill, NC, United States of America; National and Kapodistrian University of Athens, GREECE

## Abstract

**Background:**

South Africa has one of the highest rates of HIV-1 (HIV) infection world-wide, with the highest rates among young women. We analyzed the molecular epidemiology and evolutionary history of HIV in young women attending high school in rural South Africa.

**Methods:**

Samples were obtained from the HPTN 068 randomized controlled trial, which evaluated the effect of cash transfers for school attendance on HIV incidence in women aged 13–20 years (Mpumalanga province, 2011–2015). Plasma samples from HIV-infected participants were analyzed using the ViroSeq HIV-1 Genotyping assay. Phylogenetic analysis was performed using 200 *pol* gene study sequences and 2,294 subtype C reference sequences from South Africa. Transmission clusters were identified using Cluster Picker and HIV-TRACE, and were characterized using demographic and other epidemiological data. Phylodynamic analyses were performed using the BEAST software.

**Results:**

The study enrolled 2,533 young women who were followed through their expected high school graduation date (main study); some participants had a post-study assessment (follow-up study). Two-hundred-twelve of 2,533 enrolled young women had HIV infection. HIV *pol* sequences were obtained for 94% (n = 201/212) of the HIV-infected participants. All but one of the sequences were HIV-1 subtype C; the non-C subtype sequence was excluded from further analysis. Median pairwise genetic distance between the subtype C sequences was 6.4% (IQR: 5.6–7.2). Overall, 26% of study sequences fell into 21 phylogenetic clusters with 2–6 women per cluster. Thirteen (62%) clusters included women who were HIV-infected at enrollment. Clustering was not associated with study arm, demographic or other epidemiological factors. The estimated date of origin of HIV subtype C in the study population was 1958 (95% highest posterior density [HPD]: 1931–1980), and the median estimated substitution rate among study *pol* sequences was 1.98x10^-3^ (95% HPD: 1.15x10^-3^–2.81x10^-3^) per site per year.

**Conclusions:**

Phylogenetic analysis suggests that multiple HIV subtype C sublineages circulate among school age girls in South Africa. There were no substantive differences in the molecular epidemiology of HIV between control and intervention arms in the HPTN 068 trial.

## Introduction

South Africa has one of the highest rates of human immunodeficiency virus type 1 (HIV) infection in the world [1]. The highest HIV prevalence rates have been reported in the KwaZulu-Natal and Mpumalanga provinces (18% and 15.2%, respectively; ages 15–49; 2016) [2]. Adolescent girls and young women are at increased risk of HIV infection. In 2012, an estimated four million women in South Africa aged 15 and over were living with HIV/AIDS, with HIV prevalence rates of 5.6% among those aged 15–19 and 17.4% among those aged 20–24 [1]. In this region, young women acquire HIV infection earlier and have higher HIV incidence rates compared to young men [3, 4]. Several studies have evaluated HIV infection among high school students in South Africa. Studies of young women attending high-school in rural KwaZulu-Natal found higher HIV prevalence and incidence among those women than their male peers [3, 5].

Phylogenetic analysis of HIV sequences provides insights into viral transmission dynamics independent of self-reported risk behaviors and HIV prevalence data [6]. The number of available HIV sequences in public databases from sub-Saharan Africa is limited considering the size of its epidemic. Available sequence data have been used to elucidate the origin of HIV and its spread from central Africa, to identify transmission clusters, and for surveillance of HIV drug resistance [7, 8]. Phylogenetics has also been used to estimate the efficacy of interventions for HIV prevention in randomized controlled trials [9, 10], including trials evaluating antiretroviral therapy for HIV prevention [10–12].

More intensive sampling of local African epidemics is underway to gain insight into community-level transmission dynamics and to identify strategic targets for HIV prevention interventions [13]. For example, a recent phylogenetic study of young women in Kwa-Zulu Natal, South Africa showed high levels of viral diversity among and few large clusters. This study also showed that sequences from older men and young women tended to cluster, suggesting a possible role for age-disparate partnerships in the African epidemic [14]. Phylogenetic data from Uganda also show high levels of viral diversity in village communities with limited spatial clustering of incident HIV cases and local background sequences, implying geographically-dispersed transmission networks and frequent community-level viral introductions [15].

In this report, we examine molecular epidemiology and evolutionary history of HIV among adolescent girls and young women in Mpumalanga province, South Africa. Individuals 13–20 years of age were enrolled in the HIV Prevention Trials Network (HPTN) 068 study. Specifically, we sought to identify phylogenetic clusters, which are groups of genetically similar viruses, presumably close together along a transmission chain [16]. In this study, phylogenetic clusters are representations of partially sampled, indirectly linked HIV transmission chains because only women were included in the analysis [17]. HPTN 068 was conducted within the Agincourt Health and socio-Demographic Surveillance System (Agincourt HDSS) site in the rural Bushbuckridge sub-District in Mpumalanga province of South Africa [18]. In 2010–2012, HIV prevalence in the study area was over 45% among men and women who were 35–39 years old [19]. Migration for work purposes is common in this area; as many as 60% of adult men and 30% of women migrate from rural to urban areas to find work in any given year [20].

## Materials and methods

### Study cohort

HPTN 068 was a phase 3, randomized controlled trial (NCT01233531) in rural Mpumalanga province (Bushbuckridge sub-district), South Africa (enrollment period: March 2011 to December 2012) [17]. The study evaluated the effect of cash transfer for school attendance on HIV incidence among young women attending high school (enrollment age: 13–20 years). Participants were excluded if they were married or pregnant, had no parent or legal guardian living in the household, or for other reasons that might have impacted the participant’s health, well-being or study conduct. Participants were randomized 1:1 to one of two study arms: (1) 1,225 received a monthly cash transfer based on school attendance (≥80% of school days per month, intervention arm), and (2) 1,223 did not receive a cash transfer (control arm). Participants were tested for HIV infection at enrollment and annually after enrollment until the end of the trial or their expected high-school graduation date, whichever came first. The study found no significant difference in HIV incidence between study arms [17]. At the end of the main study, all eligible participants who were HIV-uninfected and agreed to participate in the follow-up study were tested for HIV infection at a post-study visit 1–2 years later. The study enrolled 81 HIV-infected and 2,448 HIV-uninfected young women in school grades 8 to 11, and followed them through their expected high school graduation date; some participants had a post-study follow-up visit 1–2 years later. Annual HIV incidence in the main study was 1.8% [17].

### HIV testing

HIV testing was performed at the study site at all visits through graduation. All samples were retrospectively tested at the HPTN Laboratory Center (Baltimore, MD, USA) to confirm HIV status. Methods used for HIV testing in the main study are described in a previous report [17]. The same methods were used in the follow-up study, with one exception: the Geenius HIV ½ Supplemental Assay (Bio-Rad Laboratories, Inc., Hercules, CA, USA) was used for retrospective confirmation of HIV seroconversion, rather than Western blot testing.

### HIV genotyping

Plasma samples from HIV-infected participants with viral loads >400 copies/mL were analyzed using the ViroSeq HIV-1 Genotyping assay, version 2.8 (Abbott Molecular, Des Plaines, IL, USA). This system generates sequences encoding HIV protease and amino acids 1–335 of HIV reverse transcriptase (1,302 base pairs, *pol* gene, corresponding to nucleotides 2252–3554 in the HXB2 K03455 reference strain). HIV drug resistance was assessed using software provided with the ViroSeq system. HIV drug resistance mutations were considered as major according to the Stanford HIV drug resistance database [21].

### Other laboratory testing

CD4 cell count testing was performed at the study sites for participants with HIV infection. HIV viral load testing was performed retrospectively at the HPTN Laboratory Center using the RealTime HIV-1 Viral Load assay (Abbott Molecular, Des Plaines, IL). Pregnancy history was collected as described [22]. Herpes simplex virus type 2 (HSV-2) testing was performed as described [17].

### Phylogenetic analysis

HIV subtyping was performed using the REGA HIV Subtyping tool v3.0 [23]. Subtyping results were confirmed with COMET HIV-1 and by approximately maximum-likelihood phylogenetic methods using FastTree v2.1.9 [24] with HIV subtype reference sequences from the Los Alamos National Laboratory’s (LANL) HIV Sequence Database [25]. Phylogenetic analysis was performed using HIV *pol* sequences from HPTN 068 participants and HIV *pol* reference sequences. HIV reference sequences from South Africa were obtained from the LANL HIV Sequence Database [25]. Reference sequences were selected using the following search criteria: subtype C; genomic region: positions 2252 to 3554; geographic region: South Africa (country code ZA). One reference sequence per individual in LANL was included in the analysis. Sequences were aligned using MAFFT software [26]; minimal manual editing was performed. Recombination analyses were conducted for reference and study sequences using RDP [27], Maxchi [28], Chimaera [29], Bootscan [30], and Siscan [31] integrated into Recombination Detection Program v4 (RDP4) [32], and suspected inter-subtype recombinant viruses were excluded from subsequent analysis. RDP4 software was run using default settings, with the following exception: the window size was set to 60 for RDP, 120 for Maxchi and Chimera, and 500 for Bootscan and Siscan tests. Approximately maximum-likelihood phylogenetic trees were reconstructed using FastTree v2.1.9 [24] with the GTR+CAT model of nucleotide substitution and Shimodaira-Hasegawa (SH) test for clade support. We also reconstructed phylogenetic trees using RAxML v8.2.10 [33] with the GTR+CAT model of nucleotide substitutions and 1,000 bootstrap iterations for confirmation of clusters. All trees were visualized with FigTree v1.4.2 [34]. Molecular genetic networks were also constructed using the HIV-TRACE software as previously described [35]. Transmission clusters were identified from phylogenetic trees using Cluster Picker v1.2.3 [36] and from phylogenetic networks using HIV-TRACE [37]. Clusters were defined using a 4.5% maximum genetic distance threshold between all sequences in the cluster and as well as a 90% minimum clade support threshold with the Cluster Picker software. Sensitivity analyses were conducted at 2.5% and 1% genetic distance thresholds. For HIV-TRACE, a genetic distance threshold of 2.5% based on genetic pairwise distances was used. These threshold values have been previously applied in other studies of HIV transmission clusters [14, 16, 36]. Phylogenetic trees were displayed using Interactive Tree of Life (iTOL) v4.1 [38].

### Phylodynamics of HIV subtype C

To explore the origination date and genetic diversity of 200 HIV subtype C *pol* gene study sequences, we conducted a Bayesian Markov Chain Monte Carlo (MCMC) phylogenetic analysis using the BEAST v1.8.4 software [39, 40]. Analyses were performed under the HKY+Γ4 and GTR+Г4 substitution models and log-normal uncorrelated relaxed molecular clock model. A Gaussian Markov random field (GMRF) Bayesian Skyride coalescent model was used to reconstruct viral population dynamics. Two independent runs were performed for 5x10^7^ steps with sampling every 1,000 generations. Convergence was assessed using Tracer v1.6 [41]. The cut-off for effective sample size (ESS) >200 was used for all parameters.

### Statistical analysis

Logistic regression analysis was used to estimate associations between demographic and epidemiological characteristics. Variable assessed included age and school grade at enrollment, study arm, timing of HIV infection (enrollment, main study, and follow-up study), viral load and CD4 cell count at the first HIV-positive visit, the presence of the major drug resistance mutations, HSV-2 infection, pregnancy history, and the probability of study sequence clustering. We also assessed excess co-clustering by study arm and other categorical epidemiological characteristics by analyzing the probability of two sequences sharing a genetic cluster (<4.5 genetic distance threshold) also shared the same characteristic relative to the probability that a random pair of sequences from two girls which were not clustered also shared that same characteristic [15]. In the case of no excess co-clustering, we expect this relative probability to be one. Analyses were performed using R v3.3.2 [42] “base” and “ape” packages.

### Nucleotide sequence accession numbers

Study sequences were submitted to GenBank under accession numbers KY883695-KY883762, KY888784-KY888875, KY921717-KY921757.

### Ethics statement

Written consent for participation in the HPTN 068 study was provided by all study participants and their parents/guardians. The study was approved by ethical review board at the University of the Witwatersrand and the University of North Carolina.

## Results

### Summary of study sequences

**Fig 1:**
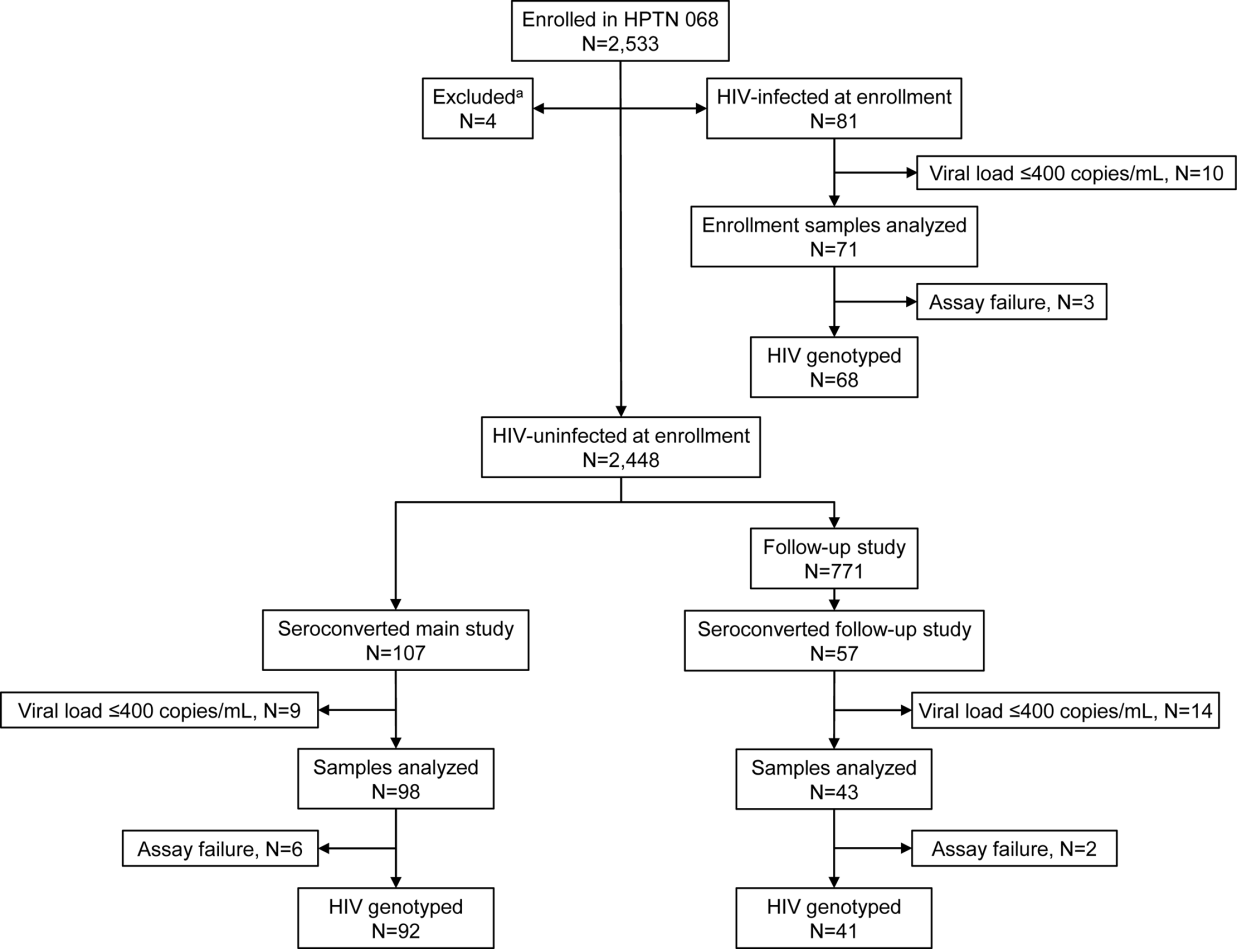
HPTN 068 study cohort flowchart. The figure provides an overview of the study cohort, including the number of samples tested and the number of HIV genotyping results obtained in each participant group: infected at enrollment, infected during the main study (between enrollment and their expected graduation date), or infected during the follow-up study (after their expected graduation date). Abbreviations: mL: milliliter. Footnote for Fig 1: ^a^Four participants were excluded due to unknown HIV status.

In HPTN 068, 245 HIV infections were documented: 81 participants were HIV infected at enrollment; 107 acquired HIV infection in the main study (between the enrollment visit and final main study visit), and 57 acquired HIV infection in the follow-up study (between the final main study visit and the post-study visit) (Fig 1). Plasma samples with HIV viral loads >400 copies/mL were available from 212 (86.5%) of the 245 HIV-infected participants (one sample per participant, collected at enrollment or the first HIV-positive visit; 33 samples had viral loads ≤400 copies/mL). HIV genotyping was successful for 201 (94.8%) of the 212 samples (68 who had HIV infection at enrollment; 92 who acquired HIV infection during the main study, 41 who acquired HIV infection during the follow-up study). Two hundred (99.5%) of the 201 sequences were HIV subtype C. One sequence was HIV subtype A; this sequence was excluded from further phylogenetic analysis. Drug resistance mutations identified using the ViroSeq HIV-1 Genotyping system were detected in 20 (10%) of the 200 sequences. At least one major non-nucleoside reverse transcriptase inhibitor (NNRTI)-resistance mutation (K103N, V106M, Y181C, G190A/S) was detected in 19 sequences; five of those sequences also had the M184V nucleoside reverse transcriptase inhibitor (NRTI)-resistance mutation. One sample had the L210W NRTI resistance mutation.

### Identification of phylogenetic clusters

**Fig 2:**
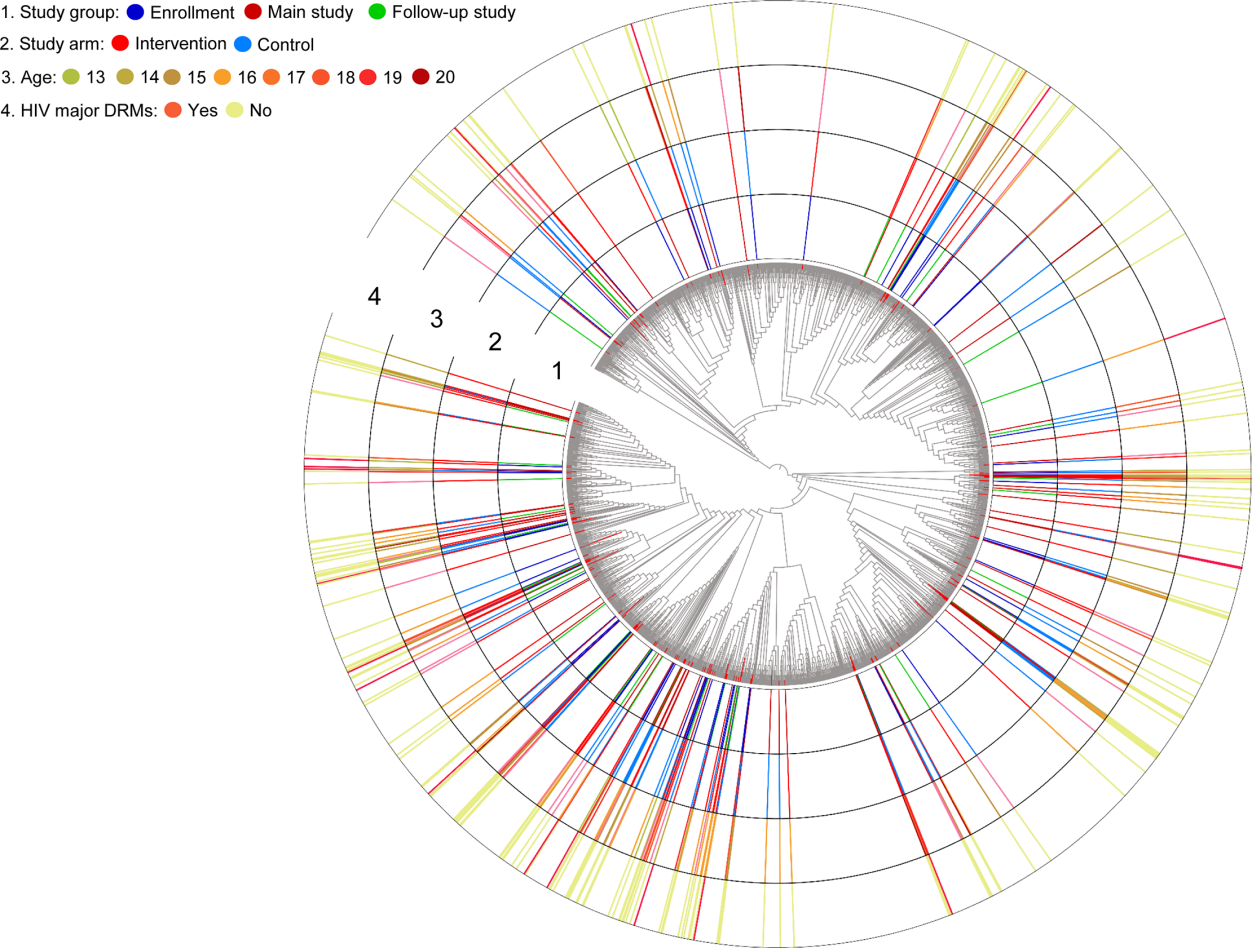
Approximately maximum-likelihood phylogenetic tree of 2,494 HIV subtype C *po*l sequences. Study sequences are indicated in the phylogeny with red branches. Abbreviation: DRM: drug resistance mutation.

**Fig 3:**
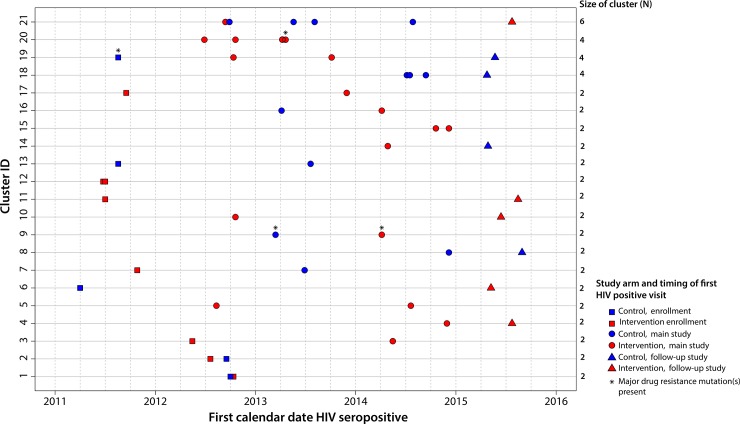
Transmission clusters from approximately maximum-likelihood phylogenetic tree. Phylogenetic clusters were detected in the approximately maximum-likelihood phylogenetic tree (FastTree) at a 4.5% genetic distance threshold. Each row represents one of the twenty-one phylogenetic clusters. Symbols representing participants in clusters are colored in red (intervention) or blue (control). Symbols represent the timing of participants’ first positive HIV test (enrollment, main study, follow-up study). Data from participants are shown on the x-axis (calendar time) according to the date of their first HIV-positive visit in the study. Drug resistant viruses are denoted with an asterisk.

Phylogenetic analysis was performed using 200 *pol* sequences from the study participants and 2,294 HIV subtype C reference sequences from South Africa; four reference sequences were excluded from the analysis (three were identified as inter-subtype recombinant; one had a high level of sequence ambiguity, >5%) (Fig 2). Overall, median pairwise genetic distance between all *po*l gene sequences including references was 6.4% (interquartile range [IQR]: 5.7–7.2%). Using Cluster Picker software with genetic distance and clade support thresholds of 4.5% and 90% respectively, 52 sequences (26% of total) from study participants clustered with one or more sequences from other study participants (Table 1). No large clusters (over 10 individuals per cluster) were identified. Most of the clusters were pairs (n = 17); clusters of four participants (N = 3) and six participants (N = 1) were also identified. Thirteen (62%) of these 21 clusters included women who were HIV infected at enrollment (Fig 3). Median genetic pairwise distance among sequences sharing a cluster was 1.1% (IQR: 0.7–2.6%) (S1 Fig). There were no reference sequences in these clusters; however, seven women (3.5%) clustered with one or more reference sequences in other clusters. Thirty-five (67.3%) of the 52 clustered sequences were in the intervention arm and of these sequences, 18 (51.4%) clustered with control arm sequences. As expected, the number of clusters decreased when more stringent genetic distance thresholds were applied (Table 1).

**Table 1:** Phylogenetic clusters containing two or more study sequences detected using different tree reconstruction and cluster detection algorithms.

Phylogenetic tree reconstruction method	Cluster detection software	Clade support threshold	Maximum genetic distance threshold	Total number of clusters detected	Cluster size distribution (N)^a^
FastTree	Cluster Picker	90%	4.5%	21	2 (17), 4 (3), 6 (1)
RAxML	Cluster Picker	90%	4.5%	21	2 (18), 4 (2), 6 (1)
FastTree	Cluster Picker	90%	2.5%	16	2 (15), 6 (1)
RAxML	Cluster Picker	90%	2.5%	16	2 (15), 6 (1)
FastTree	Cluster Picker	90%	1.0%	6	2 (6)
RAxML	Cluster Picker	90%	1.0%	6	2 (6)
-	HIV-TRACE	-	2.5%	16	2 (15), 6 (1)

^a^Cluster size distribution (number) is shown for clusters containing two or more study sequences.

Abbreviation: N: number.

The largest cluster of six included participants who seroconverted during the main (n = 5) and follow-up (n = 1) studies. The mean genetic distance in this cluster was 0.8% with an estimated time to the most recent common ancestor (tMRCA) 2006 (95% highest posterior density [HPD]: 1988–2010). Four of the participants were in the control arm and two were in the intervention arm (Fig 3). Drug resistance mutations were not detected in any of the participants within this cluster. One cluster of four participants contained women from the control arm only. Three participants within this cluster were HIV-infected during the main study; one woman was infected during the follow-up study (Fig 3). A second cluster of four women included participants from intervention arm only (Fig 3); all four were infected during the main study and were in the same school grade (10th) at study enrollment. A single drug resistance mutation was detected in one woman. The third cluster of four women included participants from both study arms who were HIV-infected at different stages in the study (enrollment, main study, follow-up study) of the trial (Fig 3). One drug resistance mutation was detected in one woman in this cluster. Study sequences were otherwise intermingled with reference sequences from South Africa throughout the tree.

In sensitivity analyses, the vast majority of phylogenetic clusters identified with FastTree were also identified with RAxML at a genetic distance threshold of 4.5%. The same clusters of viral sequences were identified at genetic distance thresholds of 2.5% and 1% (Table 1). Identical clusters were also found using Cluster Picker and HIV-TRACE at a genetic distance threshold of 2.5% (Table 1).

Associations of demographic and clinical characteristics with HIV clustering are shown in Table 2. There was also no statistically significant excess co-clustering by study arm, drug resistance mutations detected, or any other study characteristic.

**Table 2:** Associations between demographic and epidemiological characteristics of 200 HIV-infected study participants and phylogenetic clustering (4.5% genetic distance threshold).

Characteristic	Total number; N = 200 N (%)	Clustered; N = 59 N (%)	Odds ratio (95% CI)	P-value
**Age (years)**				
*15 and younger*	69 (34.5%)	22 (37.3%)	1.0 (ref)	…
*16*	46 (23%)	13 (22%)	0.84 (0.36–1.89)	0.68
*17*	44 (22%)	11 (18.7%)	0.71 (0.3–1.64)	0.43
*18 and older*	41 (20.5%)	13 (22%)	0.99 (0.43–2.26)	0.99
**School grade**				
*8*	39 (19.5%)	9 (15.3%)	1.0 (ref)	…
*9*	41 (20.5%)	15 (25.4%)	1.92 (0.43–5.28)	0.19
*10*	71 (35.5%)	19 (32.2%)	1.22 (0.5–3.14)	0.67
*11*	49 (24.5%)	16 (27.1%)	1.62 (0.36–4.33)	0.32
**Study arm**				
*Control*	95 (47.5%)	24 (40.7%)	1.0 (ref)	…
*Intervention*	105 (52.5%)	35 (59.3%)	1.48 (0.8–2.76)	0.21
**First HIV positive test**				
*Enrollment*	68 (34%)	16 (27.1%)	1.0 (ref)	…
*Main study*	92 (46%)	32 (54.2%)	1.73 (0.87–3.27)	0.65
*Follow-up study*	40 (20%)	11 (18.6%)	1.23 (0.5–3)	0.13
**Viral load, copies/mL**				
*400–9*,*999*	61 (30.5%)	22 (37.3%)	1.0 (ref)	…
*10*,*000–99*,*000*	112 (56%)	27 (45.8%)	0.56 (0.29–1.11)	0.1
*>100*,*000*	27 (12.5%)	10 (16.9%)	1.04 (0.39–2.65)	0.93
**CD4 cell count, cells/mm**^**3,**^^**a**^				
*>500*	92 (46%)	30 (50.9%)	1.0 (ref)	…
*< = 500*	75 (37.5%)	19 (32.2%)	0.70 (0.35–1.37)	0.31
*< = 250*	15 (7.5%)	4 (6.8%)	0.75 (0.2–2.4)	0.65
**Major drug resistance mutation**				
*No*	180 (90%)	55 (93.2)	1.0 (ref)	…
*Yes*	20 (10%)	4 (6.8%)	0.57 (0.16–1.63)	0.33
**HSV-2 infection**^**b**^				
No	90 (45%)	25 (42.4%)	1.0 (ref)	…
Yes	69 (34.5%)	23 (39%)	1.3 (0.65–2.57)	0.45
**Ever pregnant**^**c**^				
No	122 (61%)	37 (62.7%)	1.0 (ref)	…
Yes	49 (24.5%)	15 (25.4%)	1.01 (0.48–2.06)	0.971

^a^CD4 cell count data was not available for 18 women

^b^HSV-2 infection status was not available for 41 women

^c^Pregnancy history was not available for 29 women.

Abbreviations: N: number; CI: confidence intervals; ref: reference.

### Phylodynamics of HIV subtype C in the trial population

**Fig 4:**
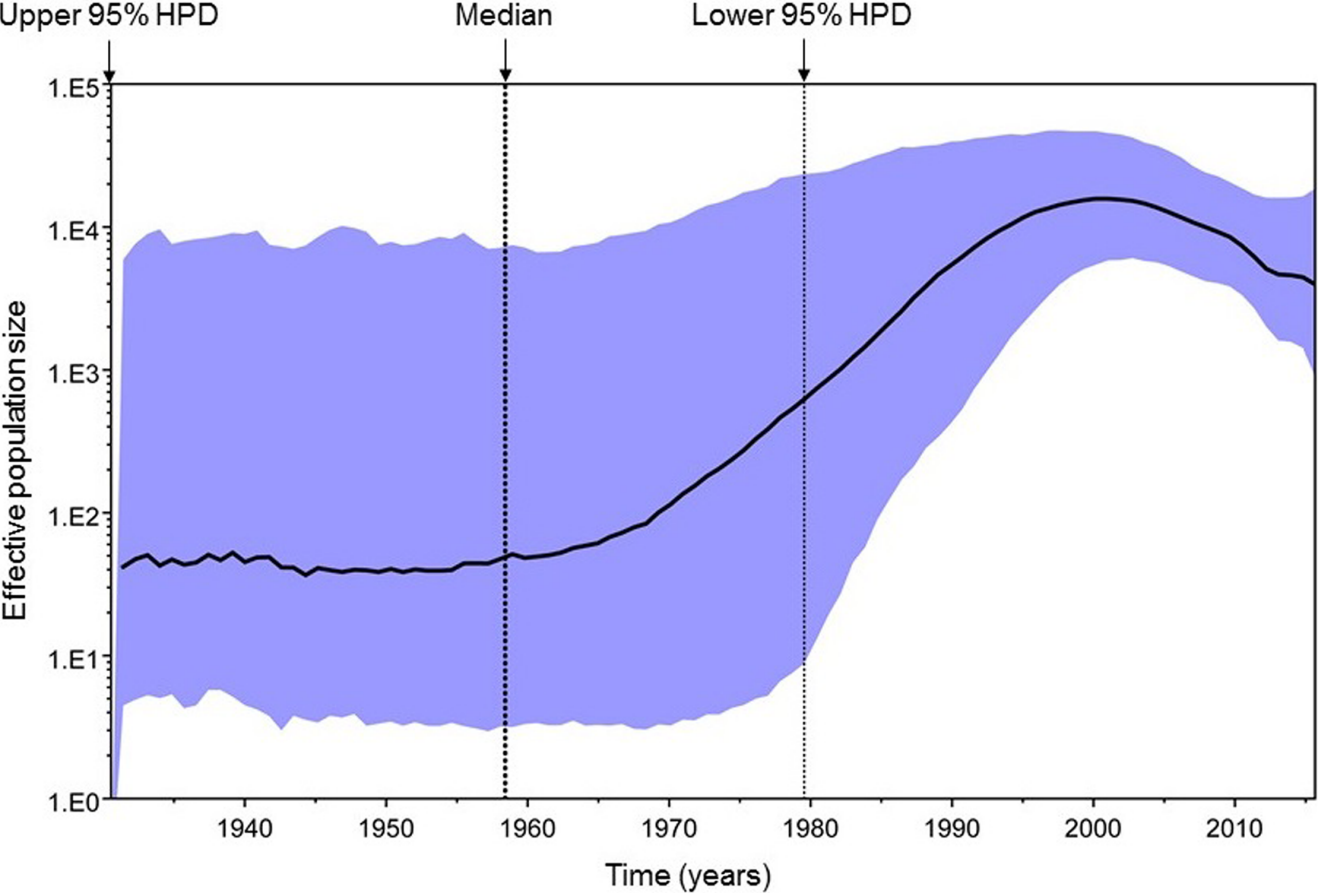
GMRF Bayesian Skyride plot of HIV subtype C. The GMRF Bayesian Skyride plot was reconstructed from the 200 *pol* gene sequences. Bold black line indicates the median effective population size through time; blue shaded area represents the 95% highest posterior density (HPD) interval. The vertical dotted lines represent the estimated date of origin (1958) of HIV subtype C, and lower and upper 95% HPD intervals (1931–1980).

Phylodynamic reconstruction of the epidemic with Bayesian MCMC analysis revealed that the date of origin of HIV subtype C circulating in the study population was 1958 (95% HPD: 1931–1980) (Fig 4). There appeared to be a rapid growth in the effective size of the viral population between the 1970s and 1990s with growth peaking in the early 2000s and declining somewhat thereafter; however, changes in effective population size over time were not statistically significant. The median estimated substitution rate (i.e., the rate at genetic differences were accrued over time) among study *pol* sequences was 1.98x10^-3^ per site per year (95% HPD: 1.15x10-3–2.81x10^-3^). The estimated dates of origin and substitution rates using HKY+G4 and GTR+G4 substitution models were highly consistent. There were no substantive differences in population size trajectories between intervention and control arms of the trial population (data not shown).

## Discussion

In this study, we analyzed indirect female-to-female HIV transmission chains among young women with HIV subtype C infection who were attending high school in rural South Africa. All but one of the 201 HPTN 068 participants with genotyping results had HIV subtype C infection; one participant had HIV subtype A infection. Phylogenetic analysis revealed small distinct HIV transmission clusters among study sequences scattered across the subset of subtype C reference sequences from South Africa. Clustering was not statistically significantly associated with demographic and select epidemiological characteristics of study participants. Results in this report and results of another study from the same region (Bushbuckridge sub-district, Mpumalanga province) [43] indicate that there are multiple HIV subtype C sublineages circulating in the population of this area.

Phylogenetics is widely used to describe evolution and origins of viruses [44], to provide information about circulating genetic variants of pathogens [44], and to identify transmission clusters [6, 8]. Each cluster is most often a representation of a partially sampled transmission chain identified based on similarity of the viral sequences [6, 45]. In the HPTN 068 trial, we found that a low proportion (26%) of participants clustered in small groups no larger than six women. Low levels of clustering between HIV infections and small cluster sizes are commonly observed in heterosexual African HIV epidemics, probably because the sampling fraction of transmission networks is low [15, 45, 46]. In this report, we sampled 2,533 individuals, which was less than 1% of the total population residing in the study area (Bushbuckridge sub-district, South Africa).

The lack of structured HIV subtype C phylogeny [47] and high genetic diversity of Southern African HIV strains has been demonstrated in previous studies [45, 48]. Phylogenetic reconstruction of subtype C infections in South Africa also suggest that there have been multiple introductions of the virus to the region [49, 50] and extensive intrasubtype recombination [51]. In our study, the median pairwise genetic distance among analyzed HIV *pol* sequences was 6.4%. Similar levels of genetic diversity were found in previous studies of HIV subtype C infection in the general population in African countries [15, 45, 48, 52].

The estimated year of HIV origin into the region was 1958, based on the subset of 200 *pol* HIV subtype C sequences. The HIV subtype C effective population size significantly increased between the 1970s and 1990s, with the peak of infections in the early 2000s. A decline of the effective population size was observed thereafter. These results are compatible with previous studies of origin and evolution of HIV subtype C viruses in South Africa [49, 50] and other countries [52, 53]. The rate of molecular evolution among HPTN 068 HIV subtype C *pol* sequences was estimated as 1.98x10^-3^ per site per year. Similar mutation rates in the *pol* gene were reported previously for HIV subtypes C [50, 53] and B [54]. High migration rates likely play a substantial role in the dynamics of the epidemic [50] and probably contribute to the high level of HIV diversity in South Africa. This report provides insight into the HIV epidemic among young women in rural South Africa who were enrolled in a randomized controlled trial. We observed high HIV prevalence and multiple HIV subtype C sublineages circulating among young women in the study. There was no evidence of distinct sub-epidemics among young women by study arm, age, school grade, or timing of infection (i.e., if they were infected at enrollment, in the main study, or in the follow-up study). There was also limited evidence for super-spreading events or large, highly connected networks similar to those observed in outbreaks of HIV among persons who inject drugs in the United States [55] or HIV transmission networks among men who have sex with men [56].

## Supporting information

S1 Fig Violin and boxplots of *pol* pairwise genetic distances. Boxplots represent the median and interquartile ranges of *pol* pairwise genetic distances obtained for participants who did or did not share a phylogenetic cluster in the approximately maximum-likelihood phylogenetic tree at a genetic distance threshold of 4.5%. The violin plots represent the distribution and density of pairwise genetic distances in each group.
(TIF)
