# Supplementary figures and images for: HIV-1 diversity among young women in rural South Africa: HPTN 068
Source: PLoS One. 2018 Jul 5;13(7):e0198999. doi: 10.1371/journal.pone.0198999 (PMC6033411; doi:10.1371/journal.pone.0198999)

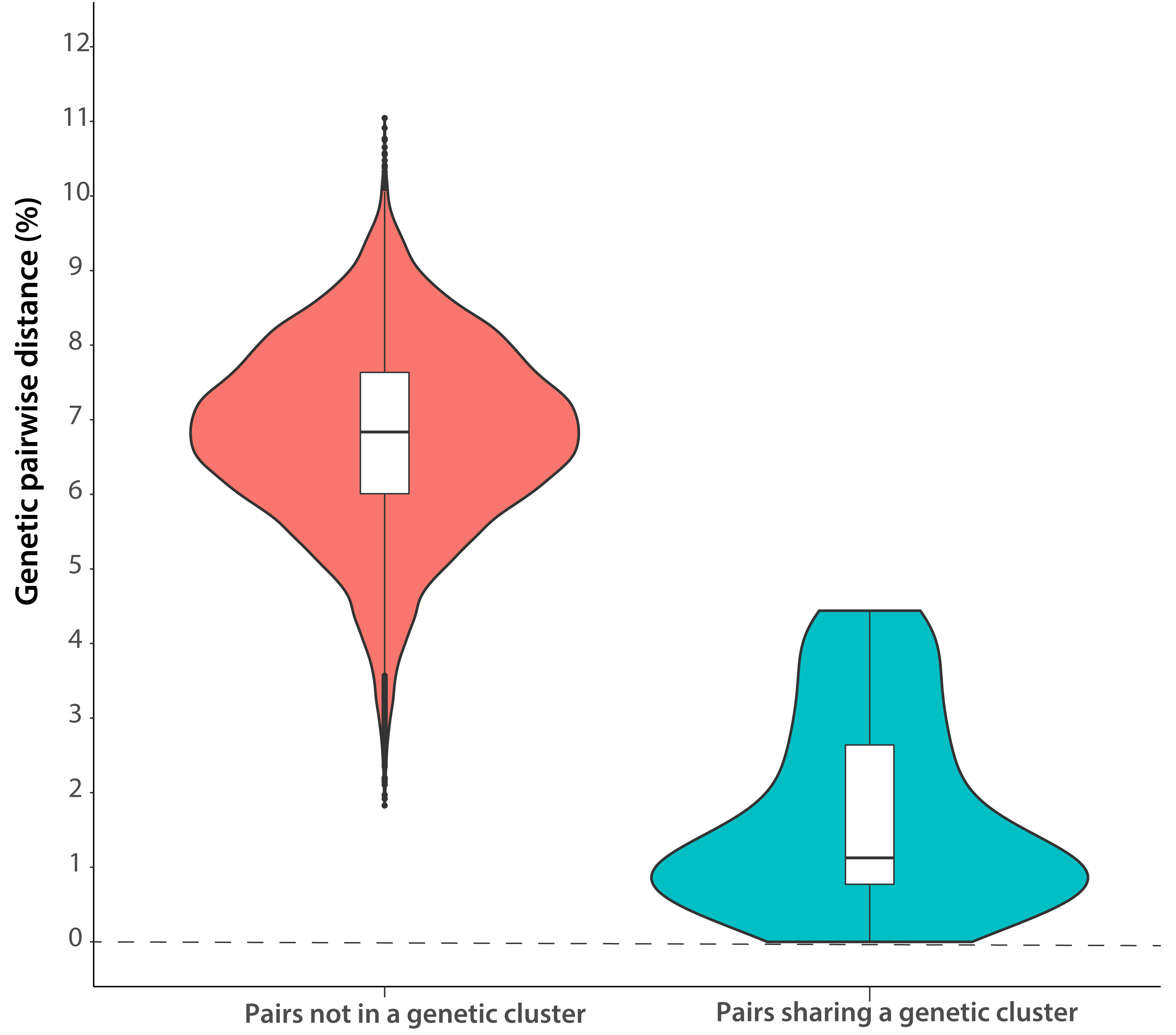

Supplement: S1 Fig — Boxplots represent the median and interquartile ranges of pol pairwise genetic distances obtained for participants who did or did not share a phylogenetic cluster in the approximately maximum-likelihood phylogenetic tree at a genetic distance threshold of 4.5%. The violin plots represent the distribution and density of pairwise genetic distances in each group. (TIF) [file pone.0198999.s001.tif]
